# Challenges with Surveillance of Healthcare-Associated Infections in Intensive Care Units in South Africa

**DOI:** 10.1155/2017/7296317

**Published:** 2017-10-12

**Authors:** Saajida Mahomed, Ozayr Mahomed, A. Willem Sturm, Stephen Knight, Prashini Moodley

**Affiliations:** ^1^School of Laboratory Medicine and Medical Sciences, College of Health Sciences, University of KwaZulu-Natal, Durban, South Africa; ^2^School of Nursing and Public Health Medicine, College of Health Sciences, University of KwaZulu-Natal, Durban, South Africa; ^3^KwaZulu-Natal Department of Health, Pietermaritzburg, South Africa

## Abstract

**Background:**

The incidence of healthcare-associated infections (HAIs) in the public health sector in South Africa is not known due to the lack of a surveillance system. We report on the challenges experienced in the implementation of a surveillance system for HAIs in intensive care units (ICUs).

**Methods:**

A passive, paper-based surveillance system was piloted in eight ICUs to measure the incidence of ventilator-associated pneumonia, catheter-associated urinary tract infection, and central line-associated bloodstream infection. Extensive consultation with the ICU clinical and nursing managers informed the development of the surveillance system. The Plan-Do-Study-Act method was utilized to guide the implementation of the surveillance.

**Results:**

The intended outputs of the surveillance system were not fully realized due to incomplete data. The organizational culture did not promote the collection of surveillance data. Nurses felt that the surveillance form added to their workload, and the infection control practitioners were unable to adequately supervise the process due to competing work demands.

**Conclusions:**

A manual system that adds to the administrative workload of nurses is not an effective method of measuring the burden of HAIs. Change management is required to promote an organizational culture that supports accurate data collection for HAIs.

## 1. Background

Healthcare-associated infections (HAIs) are a major cause of morbidity and mortality among hospitalized patients [1]. Approximately 30% of patients admitted to intensive care units (ICUs) in high income countries are affected by at least one episode of an HAI [2], and this HAI is most frequently associated with the use of invasive devices. There is a paucity of data on the burden of HAI in low-middle income countries. From the limited data available for Africa, up to 50% of patients in ICUs have been reported to acquire an HAI [3], and low-middle income countries have a much higher burden of device-associated infections.

A systematic review of HAIs in high income countries from 1995 to 2010 showed that the pooled cumulative incidence density of ventilator-associated pneumonia (VAP) was 7.9 (95% CI 5.7–10.1) per 1000 ventilator days, 4.1 (95% CI 3.7–4.6) per 1000 urinary catheter days for catheter-associated urinary tract infections (CAUTI), and 3.5 (95% CI 2.8–4.1) per 1000 central line days for central line-associated blood stream infections (CLABSI) [4]. In contrast, the burden of HAIs in low-middle income countries is much higher. During the same review period, data from low-middle income countries showed the pooled cumulative incidence density of VAP was 23.9 (95% CI 20.7–27.1) per 1000 ventilator days, 8.8 (95% CI 7.3–10.4) per 1000 urinary catheter days for CAUTI, and 12.2 (95% CI 10.5–13.9) per 1000 central line days for CLABSI [4].

The burden of HAIs is largely unknown in the public health sector in South Africa. A pilot prevalence study conducted in six hospitals in 2005 (four in the public sector and two in the private sector) for urinary tract infections, surgical site infections, primary bloodstream infections, and pneumonia showed that the combined prevalence for these four HAIs was 9.7% [5]. Data obtained from the surveillance of device-associated infections at an ICU in a private hospital in Johannesburg showed the mean VAP rate in 2014 of 6.5 per 1000 ventilator days (95% CI 0.2–13.0) and mean CAUTI rate of 1.3 per 1000 catheter days compared favourably to high income countries [6]. Whilst hospitals in South Africa's private health sector are comparable to hospitals in high income countries, this is not the case for the public health sector. The resource-constrained hospitals in the public health sector in South Africa are more comparable to hospitals in low-middle income countries. The data on HAI from a private hospital is therefore not reflective of the burden of HAI in the public health sector. A small study conducted over a six-month period in two public sector ICUs in eThekwini Health District reported a VAP incidence density of 9.9 per 1000 ventilator days, with 25% of the 32 patients acquiring a VAP during the study period [7]. Although the authors state that the 25% is comparable to other studies, there is no discussion around the incidence density of VAP which is much higher than that reported in the systematic review by the World Health Organization [4].

Healthcare-associated infections can result in a prolonged hospital stay, long-term disability, increased resistance of microorganisms to antimicrobial agents, increased risk of mortality, and a massive additional financial burden for the health system and for patients and their families [4].

The value of HAI surveillance together with appropriate infection control activities was established almost four decades ago in the Study on the Efficacy of Nosocomial Infection Control where it was demonstrated that hospitals without surveillance systems had increased HAI rates [8]. A decrease in the incidence of HAIs was also found following the establishment of surveillance systems in Germany, the Netherlands, and France [9–11].

Prevention of HAIs is one of the six quality priorities documented in the National Core Standards for Health Establishments in South Africa [12]. These guidelines specify that an infection prevention and control program must be in place to prevent HAIs, and one of the measures of this standard is the presence of a formal system to monitor infection prevention and control and ensure that appropriate actions are taken to minimise infection rates (Table 1). Although numerous improvements have been made in infection prevention and control programs, the implementation of a surveillance system for the detection of HAIs has lagged behind.

Our aim was to pilot a standardized paper-based surveillance system to obtain a baseline measurement of the incidence of HAIs in ICUs and to test the feasibility of implementing such a surveillance system for HAIs in ICUs. In this paper, we report on the implementation and challenges with implementing a paper-based surveillance system for HAIs in ICUs.

## 2. Methods

### 2.1. Study Setting

The surveillance was implemented in six ICUs in the public sector and two ICUs in the private sector in the eThekwini Health District which is the most populous district and has the largest number of hospitals and ICUs in the province of KwaZulu-Natal. Each ICU has a nursing manager, and in the public sector a clinical manager as well. Patients in the ICUs are managed by the relevant specialist clinicians, and some ICUs have critical care physicians.

### 2.2. Design and Implementation of the Surveillance System

**Figure 1:**
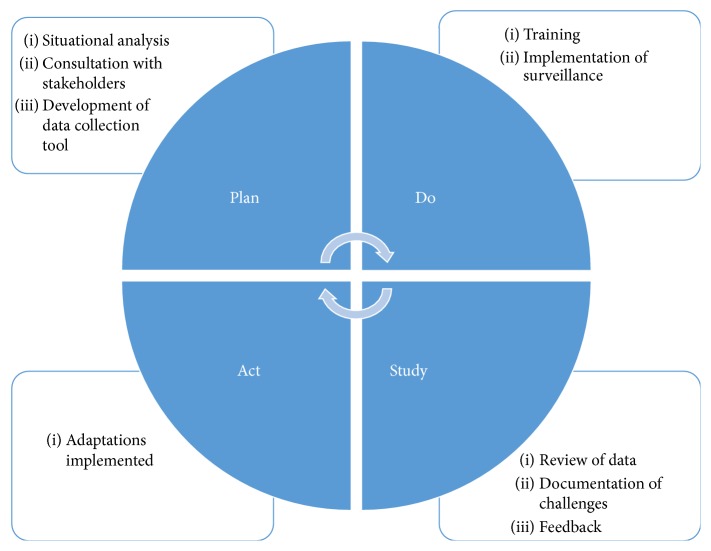
Adapted Plan-Do-Study-Act cycle approach used to implement an HAI surveillance system in ICUs.

The surveillance system was designed and implemented using the Plan-Do-Study-Act quality improvement cycle (Figure 1).

#### 2.2.1. Plan Phase

A baseline situation analysis was conducted to identify the existing methods of identifying and reporting HAIs and to assess the human resource capacity of the hospitals, specifically of the ICUs, for conducting surveillance of HAIs. After initial discussions with senior management at each hospital, there was extensive consultation with key stakeholders that included the ICU nursing and clinical manager and the hospital infection control practitioner. It was established that there was no surveillance in place for the measurement of the incidence of HAIs in the ICUs. The current method of diagnosis and reporting of an HAI in all the ICUs was through the microbiologist who would notify the attending clinician when a patient specimen yielded a positive culture. There was no link to the number of device days related to the patient specimen.

*Surveillance Tool*. A data collection tool was developed based on the minimum data required to measure the incidence of VAP, CLABSI, and CAUTI. The data elements included basic demographic data, dates of insertion and removal of each of the devices, and to where the patient was discharged. Due to perceived difficulty of having all the clinicians working in the ICUs to implement standardized definitions for each of the HAIs, it was decided that, on the tool, it would reflect whether or not the patient had developed either a pneumonia, septicemia, or urinary tract infection, and the principal investigator together with the ICU clinical manager or attending clinician would retrospectively review the patient files and assess whether or not the patient had met the National Health and Safety Network criteria for each of the HAIs. The clinical and nursing managers approved the tool prior to implementation.

#### 2.2.2. Do Phase

*Data Collection*. Training sessions were held with the infection control practitioners and nursing staff at each ICU. It was agreed that the surveillance forms would be completed prospectively where the attending nurse for each patient was to complete the relevant details for the patient daily. The date of commencement for data collection was agreed upon at each ICU and the principal investigator's contact details were made available. The infection control practitioner agreed to oversee the data collection on an ad hoc basis and to assist with queries.

#### 2.2.3. Study Phase

The principal investigator conducted fortnightly visits to each hospital, reviewed the relevant documentation, and addressed various issues raised by the nursing staff. At each of these visits, the principal investigator discussed the challenges with the hospital infection control practitioner. A formative evaluation of the implementation of the surveillance system, including identifying the strengths and challenges, was conducted using a systems approach. Feedback was provided verbally to nursing managers and the infection control practitioners on the completeness of the surveillance tool.

*Data Analysis*. Completed surveillance forms were captured electronically onto Microsoft Excel. Attempts were made to rectify data gaps via the infection control practitioner at each hospital. The number of completed forms was compared to the number of patients in the ICU each month. The incidence of each HAI was to be calculated for each ICU using the number of days on a device as the denominator and the number of each of the HAIs as the numerator.

#### 2.2.4. Act Phase

After three months of implementation of the surveillance tool, a review meeting was convened to decide on whether the surveillance system should be adopted, adapted, or abandoned.

### 2.3. Ethics

Permission was obtained from each hospital manager and the KwaZulu-Natal Provincial Department of Health. Ethics approval was obtained from the Biomedical Research and Ethics Committee at the University of KwaZulu-Natal (BE53/14).

## 3. **Results**

### 3.1. Situational Analysis on HAI Surveillance

None of the ICUs had a formal system in place for the identification and reporting of HAIs. Surveillance of HAIs was done on an ad hoc basis; for a specified time periods; and only for research purposes. None of the ICUs had data available that allowed for the calculation of the incidence density of VAP, CLABSI, or CAUTI.

### 3.2. Implementation of the Surveillance System

The implementation of the surveillance system for HAIs in ICUs received strong support from the KwaZulu-Natal Department of Health Provincial Infection Prevention and Control Unit and senior management at each of the hospitals. In addition, the ICU clinical and nursing managers and infection control practitioners displayed great enthusiasm for the implementation of the surveillance system and recognized that this system would provide them with valuable information that would improve patient care.

Despite the commitment and enthusiasm of the hospital leadership about the initiation of a surveillance system for HAIs, the implementation faced numerous challenges (Table 2). The critical challenges were linked to human resources and quality of data and are further expanded on in this manuscript.

### 3.3. Insufficient Human Resources

Each ICU did not have a dedicated critical care trained physician. Although all ICUs reported to have a sufficient number of nursing staff, the full complement of nursing staff was not always on duty due to nurses being on sick leave, vacation leave, or training, and this resulted in an increased clinical workload. Only one ICU reported to have a sufficient number of critical care trained nurses. Additionally, the ICUs did not have dedicated administrative staff to assist with the data collection for the surveillance.

### 3.4. Inadequate Oversight

The nursing managers and infection control managers did not take ownership of the process and maintain adequate oversight, as was envisaged during the planning phase. Nursing managers were often inundated with their other duties including compiling reports for hospital management and they were unable to oversee the completion of the surveillance forms. The hospital infection control practitioners reported that they had too many other work responsibilities and were therefore not able to supervise the surveillance system. They further reported that all issues that pertained to hygiene in the hospital environment became their responsibility; two situations that were cited during the piloting of the surveillance were related to the kitchen in the hospital: (i) problem with rodents around the kitchen waste area and (ii) contract kitchen staff taking home leftover food from patients' meals. The infection control practitioners conveyed that these sorts of issues did not relate to their core function and compromised their role which should be more focused on the prevention of HAI among patients in the hospital.

### 3.5. Inappropriately Designed Information Technology

Four of the ICUs had an electronic patient information system and electronic hospital information system, but these systems did not allow for the collection of the data required to measure the incidence of HAIs. A lot of the patients' clinical information is entered into the patient information system in a format that does not allow for mining of the data.

### 3.6. Increased Nursing Workload

The main challenge with the implementation of the paper-based surveillance system was the increased administrative workload on the professional nurses. The nurses verbalized that they had so many forms to fill and they did not always have the time to complete an additional form that required data that was already being captured elsewhere in the patient's clinical record.

### 3.7. Deficiencies in Training

Training was provided at each ICU; however the principal investigator was unable to have direct contact with all ICU nursing staff. The nursing manager and day nursing staff at each ICU were tasked with the responsibility of cascading the training to the night nursing staff and any staff that were on leave during the training sessions. We did not develop a standard operating procedure for the surveillance system and training.

### 3.8. Lack of Standardization of the Diagnosis of HAIs

The diagnosis of an HAI was made based on the discretion of the attending clinician; and none of the ICUs were using standardized criteria to diagnose a VAP, CLABSI, or CAUTI. It was reported that the use of the Centre for Disease Control and National Health and Safety Network criteria makes the diagnosis of device-associated HAIs very challenging due to the strict criteria. One of the aspects for the diagnosis of a VAP includes the patient's ventilator parameter and this was not recorded in the patients' daily clinical records. Even in the ICUs with the electronic patient information system, it was not possible to retrospectively review patients' ventilator parameters.

### 3.9. Poor Quality of Data

Two hundred and twenty-nine surveillance forms were received from the eight ICUs over a three-month period. The proportion of forms received ranged between 50% and 100% of the patients admitted per ICU per month. The quality of data obtained via the surveillance tool was suboptimal and precluded the calculation of the incidence of HAIs. In approximately 20% (46) of the surveillance forms, the ward or hospital to which the patient was discharged following their ICU stay was not recorded. This missing data prevented follow-up of the patient for an HAI for the required 48 hours following removal of a device. In 36% (83), 28% (65), and 28% (64) of the surveillance forms, respectively, the dates of removal of the urinary catheter and central line and date the patient was taken off ventilator were omitted, compromising the calculation of device days (Table 3).

## 4. Discussion

Despite the intensive planning, stakeholder engagement, consultation and training of nursing staff, and support from management, the surveillance of HAIs in ICUs was not successful. The main reason for the failure of the surveillance could be attributed to human resource limitations. However there are other systemic issues that contributed to this problem.

The collection of surveillance data for HAIs was not seen as a priority in our ICU settings. Although the reporting of HAIs is a requirement in the National Core Standards for Health Establishments, it is not explicitly stated in the Strategic Plan for the National Department of Health [13] and reporting of HAIs is not required in the Annual Performance Plan for the Provincial Department of Health [14]. In the South African National Development Plan which outlines the country's goals to attain by 2030, two of the health priorities are improving health information systems and improving quality by using evidence [15]. The surveillance of HAIs is linked to these two priorities in that the surveillance requires strengthening of health information systems and generates information that can assist in improving the quality of care of patients. South Africa has a high burden of communicable diseases and maternal and child mortality, with KwaZulu-Natal being the epicenter of the tuberculosis and human immunodeficiency virus epidemics [16]. As a result, surveillance of HAIs is not given sufficient attention at hospitals.

The role of nurses in activities other than the direct nursing care of a patient is becoming increasingly prominent. Nurses need to play a more important role in antimicrobial stewardship activities to ensure that these activities are sustainable and cost-effective [17]. Similarly, nurses have a fundamental role in the surveillance of HAIs, in the recording and collection of data that is then collated, analyzed, and reported on to provide information that allows for nurses and doctors to improve their practice and monitor their actions related to specific HAIs. During our implementation, nurses were unable to consistently collect the data required to complete a surveillance form for each patient, and high work demands and insufficient staffing were cited as the reasons for this. The shortage of appropriately trained critical care nurses is one of the challenges facing ICUs in South Africa [18].

Clerical staff were not considered for the collection of data as not every ICU had dedicated administrative support. In addition, the surveillance required reviewing patients' clinical records for detailed clinical information which may have posed a challenge for a nonclinical personnel. In Egypt, providing an incentive for the surveillance of HAIs resulted in improving the quality of data collected and the successful measurement of the burden of HAIs. The incentives include rewarding the ward that implements surveillance of HAI successfully with a trophy or certificate. Other measures such as employing dedicated personnel to collect data and paying nurses extra to conduct surveillance of HAIs were not sustainable in this setting [19]. The use of positive recognition and acknowledgment of staff that successfully implement surveillance of HAIs within their hospital ward is something that should be considered in our healthcare setting.

The infection control practitioner plays an integral role in the surveillance of HAIs; however due to competing work demands this role is often neglected. In addition to the work demands, inadequate resources and a suboptimal ratio of infection control practitioners to patient beds also pose a challenge for the adequate surveillance of HAIs [20]. A systematic review of the challenges in hospital management, organization, and structure in the prevention of healthcare-associated infections also identified poor leadership, insufficient resources, and the low priority of infection control as obstacles to implementing surveillance [21]. It is important that the organizational culture of the hospital encourages evidence-based healthcare that is supported by the data that a surveillance system for HAIs can generate.

Electronic health information systems can enhance surveillance activities. Even though four of the ICUs in our study had electronic patient records, the hospital electronic information system did not support the implementation of surveillance of HAIs. The lack of appropriate software as a challenge in the quality of electronic health data has been documented in other studies [20, 22]. Integration of a surveillance system within the existing patient record keeping system would be an ideal solution. However, this is often not practical where paper-based systems are used as it would additionally require someone to extract the data from the clinical records.

Although comprehensive training was provided to the nursing staff, it is possible that more detailed information on the HAIs should have been included such as the prevention, diagnostic criteria for and management of VAP, CAUTI, and CLABSI, and the consequences of these HAIs to both the patient and the health system. An additional limitation is that the surveillance was piloted over a three-month period only. The successful implementation of a similar surveillance in a private ICU in Gauteng, South Africa, took six years to establish and took at least a year before data was of a quality that allowed for analysis [6]. In our study, the surveillance was being driven by the principal investigator, who was not a staff member of any of the hospitals. Although feedback was provided at regular intervals on the quality of data being collected, it was evident that hospitals need to take ownership of the surveillance. The surveillance system was not sustainable and was therefore abandoned after the pilot period. Hospitals that have successfully implemented surveillance of HAIs have had the process driven by in-house staff, usually an infection control practitioner, microbiologist, or a clinician.

## 5. Conclusion

Good surveillance data is essential to improve the quality of patient care. A manual system that is dependent on nurses to complete and adds to the administrative workload of nurses is not an effective method of measuring the burden of HAIs in ICUs. It is important to assess the workload of all staff involved in new surveillance activities prior to implementation as this would identify whether they would be able to successfully fulfil the additional surveillance duties and the need for additional staff or task shifting. Implementation of a surveillance system should be preceded by mandatory training on the importance of surveillance data to all staff in the ICU. This training should also focus on creating collaborative teams of doctors, nurses, and administrative staff that understand their roles in generating surveillance data. The importance and utility of measuring the burden of HAIs in improving infection control practices in ICUs are required to be integrated into the professional development of clinical staff. A change management strategy should be implemented to inculcate and motivate healthcare workers across all levels to consistently collect reliable data that can be used to measure and reduce the burden of HAIs. The surveillance of HAIs should be institutionalized by including such indicators in the Annual Performance Plan of the hospitals, and the reduction of HAIs should be included as a key performance indicator for clinical and nursing managers as well as the hospital executive management.

## Figures and Tables

**Table 1:** Standards and criteria relating to prevention of healthcare-associated infections.

Domains	Subdomains for patient safety, clinical governance, and care	Standards for infection prevention and control	Criteria for infection prevention and control program
(i) Patient rights	(i) Patient care	(i) *Infection prevention and control program is in place to reduce healthcare associated infections*	(i) Infection prevention and control policy outlines health establishment's approach to managing healthcare-associated infections
(ii) *Patient safety, clinical governance, and care*	(ii) Clinical management for improved health outcomes	(ii) Specific precautions to prevent the spread of respiratory infections	(ii) A qualified health professional is responsible for infection control
(iii) Clinical support services	(iii) Clinical leadership	(iii) Standard precautions to prevent healthcare-associated infections	(iii) *A formal surveillance and reporting system is in place*
(iv) Public health	(iv) Clinical risk	(iv) Strict infection control practices are observed in the designated infant feed preparation areas	(iv) A formal system is in place to monitor infection prevention and control and ensure appropriate actions are taken to minimise infection rates
(v) Leadership and corporate governance	(v) Adverse events		(v) Reporting of healthcare- associated infections and notifiable diseases
(vi) Operational management	(vi) *Infection prevention and control*		(vi) Education of staff, patients, family, and other caregivers on infection control practices
(vii) Facilities and infrastructure			

**Table 2:** Summary of challenges in the implementation of the HAI surveillance system.

Inputs	Processes	Output
Insufficient human resources	Increased nursing workload	Poor quality surveillance data
Inadequate oversight	Deficiencies in training	
Standard operating procedure not provided	Surveillance not linked to routine data collection	
Inappropriately designed information technology	Lack of standardization on the diagnosis of HAIs	

**Table 3:** Missing data from HAI surveillance forms in public ICUs, eThekwini Health District, 2014.

Missing data	*n*	%
Date patient taken off ventilator	64	27.9
Date urinary catheter removed	83	36.2
Date central line removed	65	28.3
Discharge data	46	20.1

## References

[1] Burke J. P. (2003). Infection control - A problem for patient safety. *New England Journal of Medicine*.

[2] Vincent J.-L. (2003). Nosocomial infections in adult intensive-care units. *Lancet*.

[3] Nejad S. B., Allegranzi B., Syed S. B., Ellis B., Pittet D. (2011). Health-care-associated infection in Africa: a systematic review. *Bulletin of the World Health Organization*.

[4] World Health Organization (2011). Report on the burden of endemic health care-associated infection worldwide-A systematic review of the literature.

[5] Duse DL A. G., McIlvenny G., Rahman A., Smyth E. T. M. Healthcare Associated Infection (HCAI) Prevalence Survey: the South African Pilot.

[6] Lowman W. (2016). Active surveillance of hospital-acquired infections in South Africa: Implementation, impact and challenges. *South African Medical Journal*.

[7] Behari A. A., Kalafatis N. (2015). Incidence and outcome of ventilator-associated pneumonia in Inkosi Albert Luthuli and King Edward VIII Hospital surgical intensive care units. *Southern African Journal of Critical Care*.

[8] Hughes J. M. (1988). Study on the efficacy of nosocomial infection control (Senic project): Results and implications for the future. *Chemotherapy*.

[9] Gastmeier P., Geffers C., Brandt C. (2006). Effectiveness of a nationwide nosocomial infection surveillance system for reducing nosocomial infections. *Journal of Hospital Infection*.

[10] Geubbels E. L. P. E., Nagelkerke N. J. D., Mintjes-De Groot A. J., Vandenbroucke-Grauls C. M. J. E., Grobbee D. E., De Boer A. S. (2006). Reduced risk of surgical site infections through surveillance in a network. *International Journal for Quality in Health Care*.

[11] L'Hériteau F., Olivier M., Maugat S. (2007). Impact of a five-year surveillance of central venous catheter infections in the REACAT intensive care unit network in France. *Journal of Hospital Infection*.

[12] National Department of Health. National Core Standards for Health Establishments in South Africa. Tshwane: National Department of Health; 2011

[13] South African National Department of Health. Department of Health Strategic Plan 2014/15-2018/19. In: National Department of Health, editor. Pretoria: National Department of Health; 2014

[14] KwaZulu-Natal Department of Health. Annual Performance Plan 2014/15-2016/17. Pietermartizburg: KwaZulu-Natal Department of Health; 2014

[15] National Planning Commission. National Development Plan 2030. Pretoria; 201

[16] Kasprowicz V. O., Achkar J. M., Wilson D. (2011). The tuberculosis and HIV epidemic in South Africa and the kwazulu-natal research institute for tuberculosis and HIV. *Journal of Infectious Diseases*.

[17] Edwards R., Drumright L., Kiernan M., Holmes A. (2011). Covering more territory to fight resistance: Considering nurses' role in antimicrobial stewardship. *Journal of Infection Prevention*.

[18] Matlakala M. C., Bezuidenhout M. C., Botha A. D. (2014). Challenges encountered by critical care unit managers in the large intensive care units. *Curationis*.

[19] Kholy J. E. A success story of surveillance in a resource-limited country.

[20] Atreja A., Gordon S. M., Pollock D. A., Olmsted R. N., Brennan P. J. (2008). Opportunities and challenges in utilizing electronic health records for infection surveillance, prevention, and control. *American Journal of Infection Control*.

[21] Zingg W., Holmes A., Dettenkofer M. (2015). Hospital organisation, management, and structure for prevention of health-care-associated infection: a systematic review and expert consensus. *The Lancet Infectious Diseases*.

[22] Theobald D. (2004). The Road to Health Data Equity. *Harvard International Review*.

